# 6-Fluoro­indan-1-one

**DOI:** 10.1107/S1600536814015049

**Published:** 2014-07-02

**Authors:** Benjamin R. Slaw, Joseph M. Tanski

**Affiliations:** a Department of Chemistry, Vassar College, Poughkeepsie, NY 12604, USA

**Keywords:** crystal structure

## Abstract

The title compound, C_9_H_7_FO, crystallizes with two independent mol­ecules in the asymmetric unit, in which corresponding bond lengths are the same within experimental error. The five-membered ring in each molecule is almost planar, with r.m.s. deviations of 0.016 and 0.029 Å. In the crystal, mol­ecules form sheets parallel to (1 0 0) *via* C—H⋯O and C—H⋯F inter­actions with F⋯F contacts [3.1788 (16) and 3.2490 (16) Å] between the sheets.

## Related literature   

For the synthesis of 6-fluoroindan-1-one, see: Cui *et al.* (2004 ▶) and for its use in synthesis, see: Musso *et al.* (2003 ▶); Ślusarczyk *et al.* (2007 ▶); Yin *et al.* (2013 ▶). For the structure of the parent comound, 1-indanone, see: Morin *et al.* (1974 ▶) and Ruiz *et al.* (2004 ▶), the later containing a detailed analysis of the hydrogen bonding. For a related isomeric structure, 5-fluoroindan-1-one, see: Garcia *et al.* (1995 ▶). For more information on C—H⋯*X* inter­actions, see Desiraju & Steiner (1999 ▶) and on fluorine–fluorine inter­actions in the solid state, see: Baker *et al.* (2012 ▶). For van der Waals radii, see: Bondi (1964 ▶).
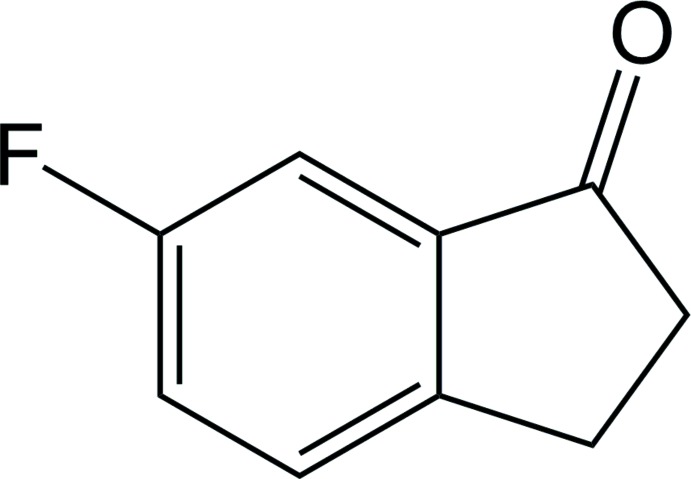


## Experimental   

### 

#### Crystal data  

C_9_H_7_FO
*M* *_r_* = 150.15
Monoclinic, 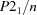

*a* = 7.1900 (4) Å
*b* = 12.4811 (6) Å
*c* = 15.8685 (8) Å
β = 99.453 (1)°
*V* = 1404.69 (13) Å^3^
*Z* = 8
Mo *K*α radiation
μ = 0.11 mm^−1^
*T* = 125 K
0.37 × 0.26 × 0.04 mm

#### Data collection  

Bruker APEXII CCD diffractometer
Absorption correction: multi-scan (*SADABS*; Bruker 2007 ▶) *T*_min_ = 0.91, *T*_max_ = 1.00
22840 measured reflections
4298 independent reflections
3345 reflections with *I* > 2σ(*I*)
*R*_int_ = 0.029

#### Refinement  

*R*[*F* ^2^ > 2σ(*F* ^2^)] = 0.041
*wR*(*F* ^2^) = 0.121
*S* = 1.03
4298 reflections
199 parameters
H-atom parameters constrained
Δρ_max_ = 0.40 e Å^−3^
Δρ_min_ = −0.21 e Å^−3^

### 

Data collection: *APEX2* (Bruker, 2007 ▶); cell refinement: *SAINT* (Bruker, 2007 ▶); data reduction: *SAINT*; program(s) used to solve structure: *SHELXS97* (Sheldrick, 2008 ▶); program(s) used to refine structure: *SHELXL2014* (Sheldrick, 2008 ▶); molecular graphics: *SHELXTL* (Sheldrick, 2008 ▶); software used to prepare material for publication: *SHELXTL*, *OLEX2* (Dolomanov *et al.*, 2009 ▶) and *Mercury* (Macrae *et al.*, 2006 ▶).

## Supplementary Material

Crystal structure: contains datablock(s) global, I. DOI: 10.1107/S1600536814015049/kj2241sup1.cif

Structure factors: contains datablock(s) I. DOI: 10.1107/S1600536814015049/kj2241Isup2.hkl

Supporting information file. DOI: 10.1107/S1600536814015049/kj2241Isup3.cml

CCDC reference: 1010372

Additional supporting information:  crystallographic information; 3D view; checkCIF report

## Figures and Tables

**Table 1:** Hydrogen-bond geometry (Å, °)

*D*—H⋯*A*	*D*—H	H⋯*A*	*D*⋯*A*	*D*—H⋯*A*
C5—H5⋯O1^i^	0.95	2.47	3.3873 (14)	161
C14—H14⋯O2^ii^	0.95	2.65	3.5107 (14)	150
C2—H2*B*⋯F2^iii^	0.99	2.46	3.2062 (13)	132
C6—H6⋯O2^iv^	0.95	2.65	3.5338 (14)	154
C11—H11*B*⋯O1^v^	0.99	2.52	3.3348 (13)	140
C15—H15⋯F1^vi^	0.95	2.52	3.3664 (13)	148

Symmetry codes: (i) 
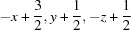
; (ii) 
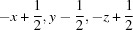
; (iii) 
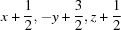
; (iv) 
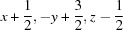
; (v) 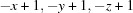; (vi) 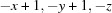.

**Fig. 1.**
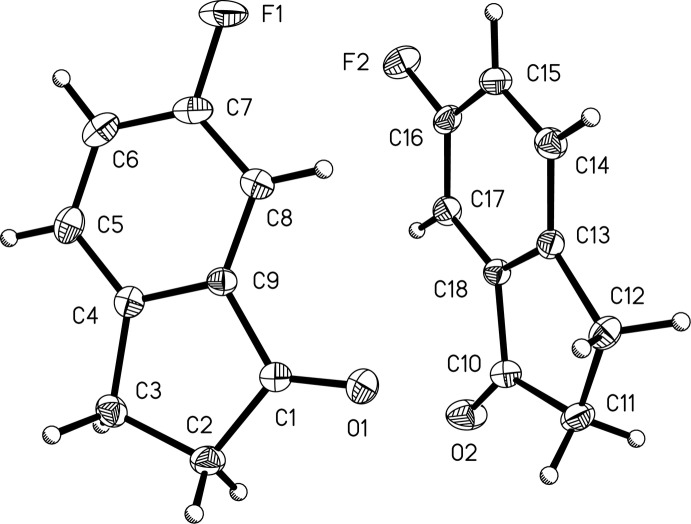
A view of the two independent molecules of the title compound, with atom numbering scheme. Displacement ellipsoids are shown at the 50% probability level.

**Fig. 2.**
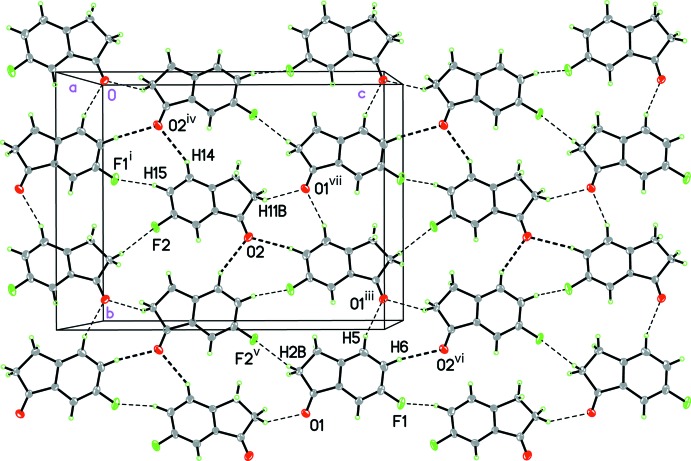
A view of the C—H···O and C—H···F interactions in the packing of 6-fluoroindan-1-one forming a sheet parallel to the 1 0 0 plane. Displacement ellipsoids are shown at the 50% probability level. Symmetry codes: (i) -x + 1, -y + 1, -z; (iii) -x + 3/2, y + 1/2, -z + 1/2; (iv) -x + 1/2, y - 1/2, -z + 1/2; (v) x + 1/2, -y + 3/2, z + 1/2; (vi) x + 1/2, -y + 3/2, z - 1/2; (vii) -x + 1, -y + 1, -z + 1.

**Fig. 3.**
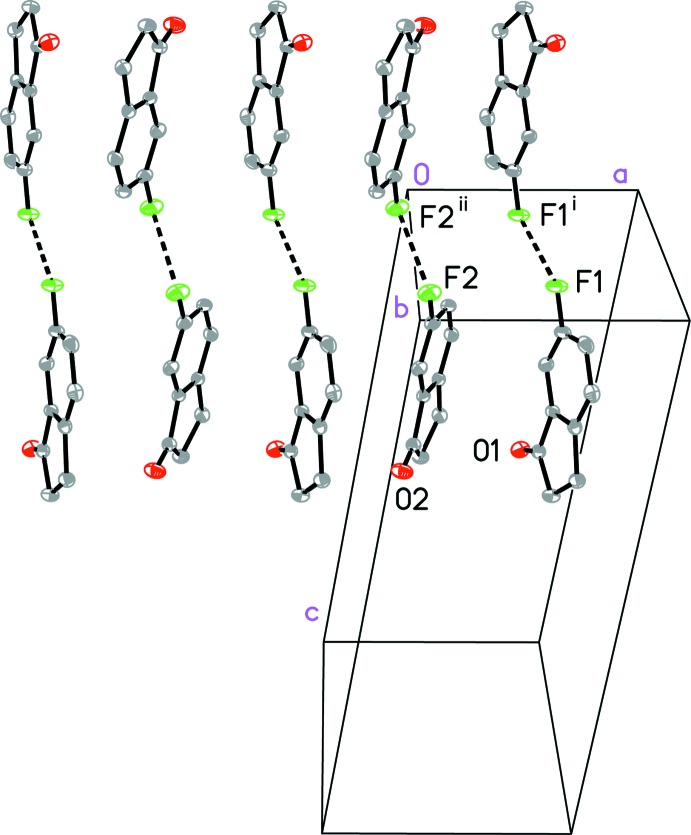
A view of the intermolecular F···F interactions in the packing of 6-fluoroindan-1-one. Distances F1···F1i 3.1788 (16) Å, F2···F2ii 3.2490 (16) Å. Displacement ellipsoids are shown at the 50% probability level; hydrogen atoms removed for clarity. Symmetry codes: (i) -x + 1, -y + 1, -z; (ii) -x, -y + 1, -z.

## supplementary crystallographic information

### S1. Comment

The titular compound 6-fluoroindan-1-one may be synthesized by the Tb(OTf)_3_-catalyzed cyclization of 3-(4-fluorophenyl)propanoic acid (Cui *et al.*, 2004). The substance has found laboratory applications in the synthesis of α-arylated compounds (Yin *et al.*, 2013), the synthesis of ethyl 2-(6-fluoro-1-hydroxy-1-indanyl)acetate, a potent muscle relaxant derivative (Musso *et al.*, 2003), and in the creation of methylene-bridged biologically active pteridine derivatives for potential hepatitis C treatments (Ślusarczyk *et al.*, 2007). The crystal structure of the parent compound, 1-indanone, has been reported previously (Morin *et al.*, 1974; Ruiz *et al.*, 2004), as has the structure of an isomer of the title compound, 5-fluoroindan-1-one (Garcia *et al.*, 1995).

The titular compound crystallizes with two molecules of 6-fluoroindan-1-one in the asymmetric unit (Figure 1). The carbonyl C—O bond lengths of 1.2172 (13) and 1.2179 (13) Å, as for the other bond lengths, are the same within the experimental error between the two independent molecules. These carbonyl C—O bond lengths are similar to those found in the structure of the parent comound, 1-indanone, 1.217 (2) Å (Ruiz *et al.*, 2004), and in the structure of the isomeric compound 5-fluoroindan-1-one, 1.218 (2) Å (Garcia *et al.*,1995). The C—F bond lengths in 6-fluoroindan-1-one, 1.3592 (12) and 1.3596 (11) Å, are also very similar to that found in the structure of the isomeric compound 5-fluoroindan-1-one, 1.354 (2) Å.

The molecules pack together in the solid state to form a two-dimensional sheet parallel to the 1 0 0 plane *via* several intermolecular C—H···O and C—F···H interactions (Figure 2, Table 2) measuring slightly less than the sum of the van der Waals radii (Bondi, 1964). The oxygen atom in each independent molecule forms two C—H···O interactions, while each independent molecule also forms one C—F···H interaction. For a discussion of C—H···*X* interactions, see Desiraju & Steiner (1999). There are also two long F···F interactions linking the two-dimensional sheets, (Figure 3, Table 1), which are somewhat longer than the sum of the van der Waals radii, 2.94 Å (Bondi, 1964). For a discussion of fluorine-fluorine interactions, which can vary widely in their metrical parameters and strength, see Baker *et al.* (2012).

### S2. Experimental

Crystalline 6-fluoroindan-1-one (I) was purchased from Aldrich Chemical Company, USA.

### S3. Refinement

All non-hydrogen atoms were refined anisotropically. Hydrogen atoms on carbon were included in calculated positions and refined using a riding model at C–H = 0.95 and 0.99 Å and *U*_iso_(H) = 1.2 × *U*_eq_(C) of the aryl and methylene C-atoms, respectively. The extinction parameter (EXTI) refined to zero and was removed from the refinement.

### Crystal data

**Table:**

C_9_H_7_FO	*F*(000) = 624
*M**_r_* = 150.15	*D*_x_ = 1.420 Mg m^−^^3^
Monoclinic, *P*2_1_/*n*	Mo *K*α radiation, λ = 0.71073 Å
*a* = 7.1900 (4) Å	Cell parameters from 9796 reflections
*b* = 12.4811 (6) Å	θ = 2.6–30.5°
*c* = 15.8685 (8) Å	µ = 0.11 mm^−^^1^
β = 99.453 (1)°	*T* = 125 K
*V* = 1404.69 (13) Å^3^	Plate, colourless
*Z* = 8	0.37 × 0.26 × 0.04 mm

### Data collection

**Table:**

Bruker APEXII CCD diffractometer	4298 independent reflections
Radiation source: fine-focus sealed tube	3345 reflections with *I* > 2σ(*I*)
Graphite monochromator	*R*_int_ = 0.029
Detector resolution: 8.3333 pixels mm^-1^	θ_max_ = 30.5°, θ_min_ = 2.1°
φ and ω scans	*h* = −10→10
Absorption correction: multi-scan (*SADABS*; Bruker 2007)	*k* = −17→17
*T*_min_ = 0.91, *T*_max_ = 1.00	*l* = −22→22
22840 measured reflections	

### Refinement

**Table:**

Refinement on *F*^2^	Primary atom site location: structure-invariant direct methods
Least-squares matrix: full	Secondary atom site location: difference Fourier map
*R*[*F*^2^ > 2σ(*F*^2^)] = 0.041	Hydrogen site location: inferred from neighbouring sites
*wR*(*F*^2^) = 0.121	H-atom parameters constrained
*S* = 1.03	*w* = 1/[σ^2^(*F*_o_^2^) + (0.0654*P*)^2^ + 0.2949*P*] where *P* = (*F*_o_^2^ + 2*F*_c_^2^)/3
4298 reflections	(Δ/σ)_max_ = 0.001
199 parameters	Δρ_max_ = 0.40 e Å^−^^3^
0 restraints	Δρ_min_ = −0.21 e Å^−^^3^

### Special details

**Table:**

Geometry. All e.s.d.'s (except the e.s.d. in the dihedral angle between two l.s. planes) are estimated using the full covariance matrix. The cell e.s.d.'s are taken into account individually in the estimation of e.s.d.'s in distances, angles and torsion angles; correlations between e.s.d.'s in cell parameters are only used when they are defined by crystal symmetry. An approximate (isotropic) treatment of cell e.s.d.'s is used for estimating e.s.d.'s involving l.s. planes.
Refinement. Refinement of *F*^2^ against ALL reflections. The weighted *R*-factor *wR* and goodness of fit *S* are based on *F*^2^, conventional *R*-factors *R* are based on *F*, with *F* set to zero for negative *F*^2^. The threshold expression of *F*^2^ > σ(*F*^2^) is used only for calculating *R*-factors(gt) *etc*. and is not relevant to the choice of reflections for refinement. *R*-factors based on *F*^2^ are statistically about twice as large as those based on *F*, and *R*- factors based on ALL data will be even larger.

### Fractional atomic coordinates and isotropic or equivalent isotropic displacement parameters (Å^2^)

**Table:**

	*x*	*y*	*z*	*U*_iso_*/*U*_eq_	
F1	0.57983 (11)	0.61035 (7)	0.04096 (4)	0.03306 (19)	
F2	0.08173 (11)	0.60328 (6)	0.05655 (4)	0.03227 (19)	
O1	0.58188 (12)	0.56668 (7)	0.37571 (5)	0.02425 (18)	
O2	0.10060 (13)	0.64991 (7)	0.39289 (5)	0.0290 (2)	
C1	0.63562 (14)	0.65257 (8)	0.35245 (6)	0.01661 (19)	
C2	0.69797 (16)	0.74848 (9)	0.40858 (7)	0.0209 (2)	
H2A	0.8032	0.7285	0.4543	0.025*	
H2B	0.5922	0.7756	0.4354	0.025*	
C3	0.76215 (15)	0.83443 (8)	0.35003 (7)	0.0198 (2)	
H3A	0.6904	0.9017	0.3526	0.024*	
H3B	0.8983	0.8498	0.3666	0.024*	
C4	0.72154 (14)	0.78578 (8)	0.26152 (7)	0.0170 (2)	
C5	0.74667 (15)	0.83033 (9)	0.18320 (7)	0.0217 (2)	
H5	0.7957	0.9007	0.1806	0.026*	
C6	0.69871 (16)	0.76990 (10)	0.10921 (7)	0.0238 (2)	
H6	0.7148	0.7986	0.0555	0.029*	
C7	0.62701 (15)	0.66714 (9)	0.11444 (7)	0.0218 (2)	
C8	0.60039 (14)	0.62037 (9)	0.19016 (7)	0.0188 (2)	
H8	0.5513	0.5499	0.1923	0.023*	
C9	0.64971 (14)	0.68253 (8)	0.26346 (6)	0.01567 (19)	
C10	0.14218 (14)	0.56199 (9)	0.36835 (6)	0.0184 (2)	
C11	0.19726 (16)	0.46437 (9)	0.42394 (7)	0.0214 (2)	
H11A	0.0886	0.4385	0.4493	0.026*	
H11B	0.3016	0.4821	0.4707	0.026*	
C12	0.26009 (15)	0.37845 (9)	0.36496 (7)	0.0195 (2)	
H12A	0.3964	0.363	0.381	0.023*	
H12B	0.1884	0.3112	0.3678	0.023*	
C13	0.21781 (14)	0.42705 (8)	0.27662 (6)	0.01607 (19)	
C14	0.24187 (15)	0.38210 (9)	0.19853 (7)	0.0195 (2)	
H14	0.2889	0.3112	0.196	0.023*	
C15	0.19576 (15)	0.44297 (9)	0.12451 (7)	0.0212 (2)	
H15	0.2117	0.4142	0.0707	0.025*	
C16	0.12627 (15)	0.54614 (9)	0.13004 (6)	0.0204 (2)	
C17	0.10060 (15)	0.59323 (8)	0.20572 (7)	0.0189 (2)	
H17	0.0525	0.6639	0.2079	0.023*	
C18	0.14943 (14)	0.53097 (8)	0.27900 (6)	0.01578 (19)	

### Atomic displacement parameters (Å^2^)

**Table:**

	*U*^11^	*U*^22^	*U*^33^	*U*^12^	*U*^13^	*U*^23^
F1	0.0391 (4)	0.0439 (5)	0.0154 (3)	0.0011 (3)	0.0024 (3)	−0.0091 (3)
F2	0.0434 (4)	0.0365 (4)	0.0151 (3)	−0.0035 (3)	−0.0006 (3)	0.0100 (3)
O1	0.0307 (4)	0.0212 (4)	0.0204 (4)	−0.0055 (3)	0.0027 (3)	0.0036 (3)
O2	0.0401 (5)	0.0244 (4)	0.0227 (4)	0.0082 (4)	0.0053 (3)	−0.0045 (3)
C1	0.0165 (4)	0.0173 (5)	0.0158 (4)	0.0016 (4)	0.0022 (3)	−0.0003 (3)
C2	0.0274 (5)	0.0192 (5)	0.0165 (5)	−0.0002 (4)	0.0044 (4)	−0.0029 (4)
C3	0.0217 (5)	0.0158 (5)	0.0220 (5)	−0.0010 (4)	0.0041 (4)	−0.0034 (4)
C4	0.0163 (4)	0.0160 (5)	0.0192 (5)	0.0021 (3)	0.0039 (4)	0.0005 (4)
C5	0.0210 (5)	0.0195 (5)	0.0254 (5)	0.0013 (4)	0.0067 (4)	0.0051 (4)
C6	0.0224 (5)	0.0307 (6)	0.0193 (5)	0.0050 (4)	0.0070 (4)	0.0061 (4)
C7	0.0214 (5)	0.0287 (6)	0.0149 (5)	0.0047 (4)	0.0018 (4)	−0.0040 (4)
C8	0.0184 (5)	0.0195 (5)	0.0179 (5)	0.0009 (4)	0.0013 (4)	−0.0024 (4)
C9	0.0166 (4)	0.0154 (4)	0.0151 (4)	0.0010 (3)	0.0027 (3)	−0.0008 (3)
C10	0.0187 (5)	0.0205 (5)	0.0159 (4)	0.0010 (4)	0.0026 (3)	−0.0004 (4)
C11	0.0262 (5)	0.0234 (5)	0.0147 (4)	0.0006 (4)	0.0037 (4)	0.0026 (4)
C12	0.0214 (5)	0.0191 (5)	0.0182 (5)	0.0026 (4)	0.0040 (4)	0.0048 (4)
C13	0.0156 (4)	0.0166 (5)	0.0160 (4)	−0.0011 (3)	0.0027 (3)	0.0012 (3)
C14	0.0189 (5)	0.0195 (5)	0.0206 (5)	−0.0004 (4)	0.0044 (4)	−0.0027 (4)
C15	0.0212 (5)	0.0270 (5)	0.0159 (5)	−0.0047 (4)	0.0045 (4)	−0.0039 (4)
C16	0.0209 (5)	0.0258 (5)	0.0135 (4)	−0.0047 (4)	0.0003 (4)	0.0049 (4)
C17	0.0200 (5)	0.0178 (5)	0.0178 (5)	0.0000 (4)	−0.0002 (4)	0.0029 (4)
C18	0.0157 (4)	0.0169 (5)	0.0145 (4)	−0.0007 (3)	0.0019 (3)	0.0002 (3)

### Geometric parameters (Å, º)

**Table:**

F1—C7	1.3592 (12)	C7—C8	1.3772 (15)
F1—F1^i^	3.1788 (16)	C8—C9	1.3947 (14)
F2—C16	1.3596 (11)	C8—H8	0.95
F2—F2^ii^	3.2490 (16)	C10—C18	1.4790 (14)
O1—C1	1.2172 (13)	C10—C11	1.5181 (15)
O2—C10	1.2179 (13)	C11—C12	1.5392 (15)
C1—C9	1.4802 (14)	C11—H11A	0.99
C1—C2	1.5152 (14)	C11—H11B	0.99
C2—C3	1.5387 (15)	C12—C13	1.5118 (14)
C2—H2A	0.99	C12—H12A	0.99
C2—H2B	0.99	C12—H12B	0.99
C3—C4	1.5140 (14)	C13—C18	1.3898 (14)
C3—H3A	0.99	C13—C14	1.3967 (14)
C3—H3B	0.99	C14—C15	1.3924 (15)
C4—C9	1.3905 (14)	C14—H14	0.95
C4—C5	1.4000 (14)	C15—C16	1.3892 (16)
C5—C6	1.3904 (16)	C15—H15	0.95
C5—H5	0.95	C16—C17	1.3764 (15)
C6—C7	1.3898 (17)	C17—C18	1.3946 (14)
C6—H6	0.95	C17—H17	0.95
			
F1···F1^i^	3.1788 (16)	F2···F2^ii^	3.2490 (16)
			
C7—F1—F1^i^	145.61 (8)	C8—C9—C1	127.39 (9)
C16—F2—F2^ii^	94.04 (6)	O2—C10—C18	126.26 (10)
O1—C1—C9	125.92 (9)	O2—C10—C11	126.27 (10)
O1—C1—C2	126.55 (9)	C18—C10—C11	107.46 (9)
C9—C1—C2	107.53 (8)	C10—C11—C12	106.29 (8)
C1—C2—C3	106.56 (8)	C10—C11—H11A	110.5
C1—C2—H2A	110.4	C12—C11—H11A	110.5
C3—C2—H2A	110.4	C10—C11—H11B	110.5
C1—C2—H2B	110.4	C12—C11—H11B	110.5
C3—C2—H2B	110.4	H11A—C11—H11B	108.7
H2A—C2—H2B	108.6	C13—C12—C11	104.47 (8)
C4—C3—C2	104.43 (8)	C13—C12—H12A	110.9
C4—C3—H3A	110.9	C11—C12—H12A	110.9
C2—C3—H3A	110.9	C13—C12—H12B	110.9
C4—C3—H3B	110.9	C11—C12—H12B	110.9
C2—C3—H3B	110.9	H12A—C12—H12B	108.9
H3A—C3—H3B	108.9	C18—C13—C14	119.66 (9)
C9—C4—C5	119.36 (10)	C18—C13—C12	111.56 (9)
C9—C4—C3	111.52 (9)	C14—C13—C12	128.76 (10)
C5—C4—C3	129.12 (10)	C15—C14—C13	118.83 (10)
C6—C5—C4	118.94 (10)	C15—C14—H14	120.6
C6—C5—H5	120.5	C13—C14—H14	120.6
C4—C5—H5	120.5	C16—C15—C14	119.39 (9)
C7—C6—C5	119.55 (10)	C16—C15—H15	120.3
C7—C6—H6	120.2	C14—C15—H15	120.3
C5—C6—H6	120.2	F2—C16—C17	118.56 (10)
F1—C7—C8	118.46 (10)	F2—C16—C15	117.94 (9)
F1—C7—C6	118.21 (10)	C17—C16—C15	123.51 (9)
C8—C7—C6	123.32 (10)	C16—C17—C18	115.98 (10)
C7—C8—C9	116.05 (10)	C16—C17—H17	122.0
C7—C8—H8	122.0	C18—C17—H17	122.0
C9—C8—H8	122.0	C13—C18—C17	122.62 (9)
C4—C9—C8	122.78 (9)	C13—C18—C10	109.79 (9)
C4—C9—C1	109.83 (9)	C17—C18—C10	127.58 (10)
			
O2—C10—C18—C17	−3.94 (18)	C3—C4—C5—C6	−179.93 (10)
O2—C10—C18—C13	174.94 (11)	C2—C3—C4—C9	−2.21 (11)
O2—C10—C11—C12	−173.07 (11)	C2—C3—C4—C5	177.79 (10)
O1—C1—C9—C8	1.81 (17)	C2—C1—C9—C8	−177.56 (10)
O1—C1—C9—C4	−178.32 (10)	C2—C1—C9—C4	2.31 (11)
O1—C1—C2—C3	177.04 (10)	C1—C2—C3—C4	3.48 (11)
F2^ii^—F2—C16—C17	−142.91 (9)	C18—C13—C14—C15	−0.21 (15)
F2^ii^—F2—C16—C15	37.18 (10)	C18—C10—C11—C12	6.57 (11)
F2—C16—C17—C18	−179.74 (9)	C16—C17—C18—C13	−0.78 (15)
F1^i^—F1—C7—C8	−1.14 (19)	C16—C17—C18—C10	177.97 (10)
F1^i^—F1—C7—C6	179.25 (9)	C15—C16—C17—C18	0.17 (16)
F1—C7—C8—C9	−179.49 (9)	C14—C15—C16—F2	−179.69 (9)
C9—C4—C5—C6	0.07 (15)	C14—C15—C16—C17	0.40 (16)
C9—C1—C2—C3	−3.60 (11)	C14—C13—C18—C17	0.82 (15)
C7—C8—C9—C4	0.06 (15)	C14—C13—C18—C10	−178.13 (9)
C7—C8—C9—C1	179.91 (10)	C13—C14—C15—C16	−0.38 (15)
C6—C7—C8—C9	0.10 (16)	C12—C13—C18—C17	179.76 (9)
C5—C6—C7—F1	179.42 (9)	C12—C13—C18—C10	0.81 (12)
C5—C6—C7—C8	−0.16 (17)	C12—C13—C14—C15	−178.95 (10)
C5—C4—C9—C8	−0.14 (15)	C11—C12—C13—C18	3.30 (11)
C5—C4—C9—C1	179.98 (9)	C11—C12—C13—C14	−177.87 (10)
C4—C5—C6—C7	0.07 (16)	C11—C10—C18—C17	176.42 (10)
C3—C4—C9—C8	179.86 (9)	C11—C10—C18—C13	−4.70 (12)
C3—C4—C9—C1	−0.02 (12)	C10—C11—C12—C13	−5.93 (11)

Symmetry codes: (i) −*x*+1, −*y*+1, −*z*; (ii) −*x*, −*y*+1, −*z*.

### Hydrogen-bond geometry (Å, º)

**Table:**

*D*—H···*A*	*D*—H	H···*A*	*D*···*A*	*D*—H···*A*
C5—H5···O1^iii^	0.95	2.47	3.3873 (14)	161
C14—H14···O2^iv^	0.95	2.65	3.5107 (14)	150
C2—H2*B*···F2^v^	0.99	2.46	3.2062 (13)	132
C6—H6···O2^vi^	0.95	2.65	3.5338 (14)	154
C11—H11*B*···O1^vii^	0.99	2.52	3.3348 (13)	140
C15—H15···F1^i^	0.95	2.52	3.3664 (13)	148

Symmetry codes: (i) −*x*+1, −*y*+1, −*z*; (iii) −*x*+3/2, *y*+1/2, −*z*+1/2; (iv) −*x*+1/2, *y*−1/2, −*z*+1/2; (v) *x*+1/2, −*y*+3/2, *z*+1/2; (vi) *x*+1/2, −*y*+3/2, *z*−1/2; (vii) −*x*+1, −*y*+1, −*z*+1.
